# The Direct and Indirect Roles of NF-κB in Cancer: Lessons from Oncogenic Fusion Proteins and Knock-in Mice

**DOI:** 10.3390/biomedicines6010036

**Published:** 2018-03-19

**Authors:** Tabea Riedlinger, Jana Haas, Julia Busch, Bart van de Sluis, Michael Kracht, M. Lienhard Schmitz

**Affiliations:** 1 Institute of Biochemistry, Justus-Liebig-University, D-35392 Giessen, Germany; Tabea.Riedlinger@biochemie.med.uni-giessen.de (T.R.); Jana.Haas@biochemie.med.uni-giessen.de (J.H.); Julia.Busch@biochemie.med.uni-giessen.de (J.B.); 2 Department of Pediatrics, Molecular Genetics Section, University of Groningen, University Medical Center Groningen, Antonius Deusinglaan 1, 9713 AV, Groningen, The Netherlands; a.j.a.van.de.sluis@umcg.nl; 3 Rudolf-Buchheim-Institute of Pharmacology, Justus-Liebig-University, D-35392 Giessen, Germany; Michael.Kracht@pharma.med.uni-giessen.de

**Keywords:** NF-κB, transcription, cancer

## Abstract

NF-κB signaling pathways play an important role in the regulation of cellular immune and stress responses. Aberrant NF-κB activity has been implicated in almost all the steps of cancer development and many of the direct and indirect contributions of this transcription factor system for oncogenesis were revealed in the recent years. The indirect contributions affect almost all hallmarks and enabling characteristics of cancer, but NF-κB can either promote or antagonize these tumor-supportive functions, thus prohibiting global NF-κB inhibition. The direct effects are due to mutations of members of the NF-κB system itself. These mutations typically occur in upstream components that lead to the activation of NF-κB together with further oncogenesis-promoting signaling pathways. In contrast, mutations of the downstream components, such as the DNA-binding subunits, contribute to oncogenic transformation by affecting NF-κB-driven transcriptional output programs. Here, we discuss the features of recently identified oncogenic RelA fusion proteins and the characterization of pathways that are regulating the transcriptional activity of NF-κB by regulatory phosphorylations. As NF-κB’s central role in human physiology prohibits its global inhibition, these auxiliary or cell type-specific NF-κB regulating pathways are potential therapeutic targets.

## 1. The NF-κB System

The NF-κB transcription factor system represents an archetypal signaling pathway that is evolutionary conserved, with core components occurring in insects, cnidarians, and even some unicellular species [1]. One of its key functions is the induction of the immune system in response to pathogens and inflammatory stimuli, but NF-κB is also activated by further adverse stimuli, such as DNA damage and hypoxia [2,3]. The great variety of input signals is reflected by a multitude of target genes that do not only represent inducers and effectors of the immune system, but also genes that impact on cell survival, proliferation, and differentiation [4]. A characteristic feature of NF-κB is the large variety of inducers that utilize a limited set of signal transduction molecules to create transcriptional output programs that are specifically tailored to suit the particular requirements in a specific tissue or organ. This conversion of relatively unspecific input signals to highly specific output programs is not really understood and employs components of the NF-κB core signaling pathways in conjunction with multiple auxiliary systems that confer specificity.

## 2. The Core NF-κB Activation Pathways

**Figure 1:**
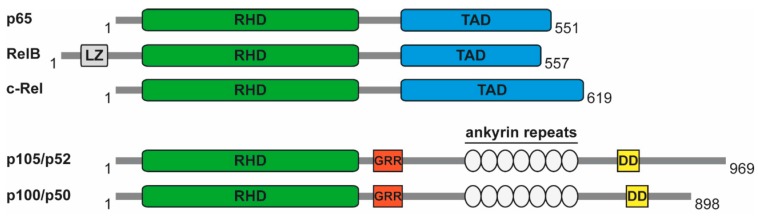
DNA-binding subunits of NF-κB. The functional domains of the five DNA-binding subunits, including the leucine zipper (LZ), the glycine-rich region (GRR), and the death domain (DD) are shown. The number of amino acids is given for the human proteins.

Five different NF-κB DNA-binding subunits share a N-terminal NF-κB/Rel homology domain (RHD), which mediates DNA-binding, dimerization, and the interaction with the inhibitory IκB proteins, as schematically shown in Figure 1. The C-terminal regions of RELA (also known as p65), RELB, and REL (also known as c-Rel) contain transactivation domains (TADs), which associate with further regulatory proteins to trigger mRNA synthesis. The family members NF-κB1 (also known as p105) and NF-κB2 (also known as p100) are precursor proteins that lack TADs but contain Ankyrin repeats in their C-terminal regions [5]. These allow for them to function as IκB proteins and to mediate protein/protein interactions. Either during translation or through phosphorylation-induced partial proteolysis, the precursors are processed to yield their DNA-binding forms p50 and p52, respectively [6]. As p50 and p52 also lack TADs, they cannot trigger transcription unless being associated with a TAD-containing NF-κB family member, suggesting that constitutive binding of p50 or p52 homodimers to κB sites represses transcription [7]. Alternatively, p50 and p52 can activate transcription upon association with IκB family members, such as Bcl3.

Activation of NF-κB can proceed by canonical, noncanonical, and the atypical NF-κB activation pathway [8]. All of the different NF-κB activating pathways ultimately result in the generation of active, DNA-binding dimers, which were beforehand retained in the cytosol of unstimulated cells by association with inhibitory IκB proteins.

In the canonical pathway, membrane anchored or cytosolic receptors sense cytokines, pathogen-associated molecular patterns (PAMPs), or damage-associated molecular patterns (DAMPs) [9]. Bound receptors self-associate to trigger signaling cascades, which proceed via posttranslational modifications (PTMs), including phosphorylation and ubiquitination. A variety of polyubiquitin chains are formed to mediate NF-κB activation including K63-branched and linear polyubiquitin chains, which are recognized and bound by chain readers such as TAB2 (TGFβ activated kinase 1/MAP3K7 binding protein 2) and NEMO (NF-κB essential modulator), respectively [10,11]. These events lead to activation of the so-called IKK (IκB kinase) complex, which is composed of the catalytic subunits IKKα and IKK in association with the scaffold protein NEMO. The activated IKKs in turn phosphorylate IκBα in order to enable its subsequent tagging with K48-branched ubiquitin chains. This allows rapid IκBα degradation by the proteasome, allowing nuclear entry, DNA-binding and transcriptional activity of the dimerized NF-κB DNA-binding subunits [8].

The noncanonical NF-κB pathway is activated in response to a distinct class of stimuli, particularly in B-cells. The activation of this pathway depends on NF-κB-inducing kinase (NIK) and IKKα, which mediate C-terminal processing of NF-κB2/p100, thereby allowing for the generation of p52/RelB dimers [12]. The noncanonical pathway requires NF-κB-inducing kinase (NIK) and IKKα, and proceeds with much slower kinetics [13].

The atypical NF-κB pathway is activated by various adverse stimuli, including DNA damage, and depends on the inducible modification of NEMO by the ubiquitin-related peptide SUMO-1 (small ubiquitin-like modifier 1). This modification process depends on the SUMO E3 ligase PIASy (protein inhibitor of activated STAT y) [14]. In addition, DNA damage leads to ATM (Ataxia Telangiectasia mutated)-mediated TRAF6 (TNF receptor associated factor 6) activation and cIAP1 (cellular inhibitor of apoptosis protein-1) recruitment, which catalyzes monoubiquitination of NEMO [15]. These modifications ultimately lead to induction of IKK activity, IκBα degradation and the release of the DNA-binding subunits.

## 3. Auxiliary NF-κB Regulating Mechanisms

Any given NF-κB activating stimulus does not only activate NF-κB, but it will necessarily trigger further signaling pathways. These additional pathways show extensive crosstalk to the NF-κB system at all levels, as elaborated in a number of excellent reviews [16,17]. Furthermore, any NF-κB activating pathophysiological situation, such as an infection, will elicit a complex mixture of NF-κB activating agents and receptors, thus leading to NF-κB activation amidst multiple co-regulated pathways that serve to dampen, trigger, or shape the NF-κB response. As a result of NF-κB activation, the DNA-binding subunits, released from their inhibitor, will bind to their cognate κB genomic binding site, which is conventionally a GGGRNNYYCC (R = purine, N = any nucleotide, Y = pyrimidine) motif [18]. Binding of NF-κB dimers to the genomic κB site is not only dictated by the accessibility of the site and by interaction with other transcription factors, such as AP1, but also by the composition of the κB binding sequence. The extensive characterization of NF-κB binding sites by EMSA-seq (electrophorectic mobility shift assays coupled to sequencing) experiments uncovered the preferred motifs for the p65/p50, p65/p65, and p65/p52 complexes [19]. For example, p65/p50 dimers preferentially bind the motif GGGGRTTTCC, while the p65/p52 heterodimer binds to the sequence NNNGGGGRYTT. Binding specificity is also achieved by the interaction of nuclear NF-κB subunits with further transcription factors, such as AP1 family members [20], Erg [21], and E2F1 [22].

Moreover, the DNA-binding capacity of NF-κB DNA-binding subunits can also be regulated in order to achieve a further level of regulation. For example, acetylation of p65 at Lys 221 causes a conformation change that favours κB DNA binding [23]. Conversely, the DNA-binding activity can be inhibited upon association of p65 with PIAS1 [24] or by PTMs, such as nitration of Tyr 66 and Tyr 152, or asymmetric dimethylation of Arg 30 [25,26]. The recent years have witnessed the identification of further PTMs at the DNA-binding subunits. The PhosphositePlus^®^ database lists 64 different modifications for the p65 subunit and comparable numbers of PTMs for p105 (61), p100 (49), c-Rel (22), and RelB (20). The DNA-binding subunits are modified by many different PTMs, including phosphorylation, ubiquitination, SUMO modification, O-GlcNAcylation, and various types of methylation. This strong increase in PTM site identification is contrasted by our limited understanding of their physiological function. Several knock-in mouse models have been generated that started to unravel the physiological roles of the individual PTMs.

## 4. Functional Analysis of p65 Phosphorylations In Vivo

**Figure 2:**
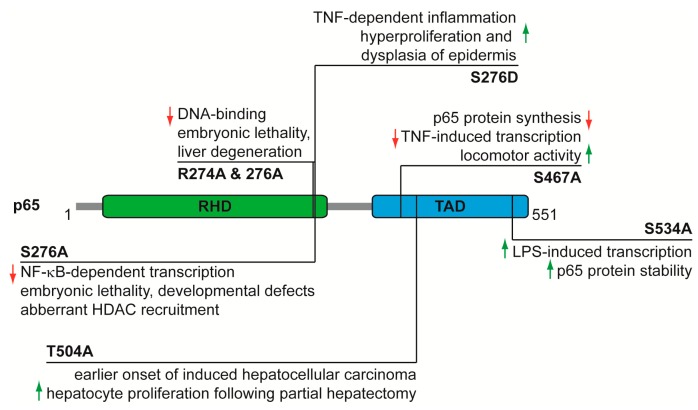
Schematic summary of the phenotypes of the p65 (RelA) knock-in mice mutated at the indicated phosphorylation sites.

The importance of individual p65 modification sites has been investigated in various knock-in mouse models, which are summarized in Figure 2. Published data are available for five different p65 knock-in mouse models and the observed range of phenotypes is remarkably broad. None of these mice that are mutated in p65 phosphorylation sites recapitulate the phenotype of RelA-deficient mice, which show embryonic lethality that is caused by massive liver degeneration with hepatocyte apoptosis [27]. The first knock-in mouse model was published almost 10 years ago by the laboratory of Sankar Ghosh [28]. In this animal, the phosphorylated Ser 276 was changed to a non-phosphorylatable Ala (p65 S276A). Cells isolated from these mice display a significant reduction of NF-κB-dependent transcription. Most of the p65 S276A knock-in embryos die at different embryonic days due to variegated developmental abnormalities in limb or eye formation, which are due to HDAC (histone deacetylase)-dependent interference with gene expression. Further experiments showed that p50/p65 S276A heterodimers cannot efficiently bind to CBP/p300 and instead recruit HDACs, resulting in aberrant repression of non-NF-κB-regulated genes through epigenetic mechanisms and the direct repression of a subset of NF-κB target genes [28]. The recruitment of HDACs requires the DNA-binding activity of p65, as demonstrated by the creation of a further knock-in mouse model where the p65 S276A mutant was additionally changed at Arg 274 to Ala, thus creating a non-DNA-binding p65 S276A/R274A double mutant [28]. These p65 S276A/R274A mice show no variegation of the phenotype, but die during embryogenesis due to massive hepatocyte apoptosis, thus resembling the phenotype of p65-deficient mice [27]. While the p65 S276A mice are phosphorylation-deficient, the p65 S276D knock-in mice express a phosphomimetic form of p65. These animals are viable, but consistent with an increased transcriptional activity of p65- show elevated expression of proinflammatory cytokines and chemokines, resulting in a systemic hyperinflammatory phenotype leading to death 8–20 days after birth. The lethality of the p65 S276D animals could be rescued by crossing with a strain lacking the TNF receptor 1 (TNFR1) [29], similar to the rescue of *RelA^-/-^*/*Tnfr^-/-^* double knockout animals [30]. However, upon aging, the p65 S276D/*Tnfr*^-/-^ mice develop chronic keratitis together with elevated expression of inflammatory cytokines in the cornea, leading to the development of a phenotype that resembles a human disease called keratoconjunctivitis sicca (“dry eyes”) [29]. In addition, p65 S276D mice show hyperproliferation and dysplasia of the mouse epidermis [31]. Several mouse mutants were generated for the analysis of phosphorylated key residues in the C-terminal TADs. Mice harboring the T505A mutation develop normally but exhibit increased hepatocyte proliferation following damage resulting from carbon tetrachloride treatment or liver partial hepatectomy [32]. The p65 T505A mice also show an earlier onset of tumorigenesis in the *N*-nitrosodiethylamine model of hepatocellular carcinoma, as consistent with previous data from reconstituted cells, suggesting that Thr 505 phosphorylation functions to suppress the tumour-promoting functions of p65 [33]. A further knock-in model addressed the relevance of Ser 534 phosphorylation, a site corresponding to the well-studied Ser 536 in human p65. The p65 S534A mice are born at normal Mendelian ratios and are healthy. Following the injection of lipopolysaccharide (LPS) they display slightly increased expression of selective NF-κB target genes and a strongly increased sensitivity to LPS-induced death [34]. The inhibitory activity of p65 Ser534 phosphorylation on NF-κB activity may also be due to a decreased half-life of the p65 protein, which is a feature that can be regulated by phosphorylation at Ser 536 and also at Ser 468 [35,36,37]. A regulatory effect of Ser 468 phosphorylation on the total amount of p65 was also observed in mice where Ser 467 (the mouse homolog of human p65 Ser 468) was mutated to Ala. This substitution causes reduced de novo synthesis of the p65 protein by an unexplored mechanism. The reduced p65 levels likely contribute to diminished TNF-induced expression of a selected group of NF-κB dependent target genes and also increased TNF-triggered apoptosis [38]. A serendipitous observation was the reduced weight gain in male p65 S467A mice. Feeding a high fat diet resulted in a strong body weight increase in wildtype animals, while male p65 S467A mice were partially protected from an increase in body weight. This phenotype is likely due to the elevated locomotor activity of these animals, but it is currently completely unclear why this phenotype only occurs in male mice [38]. It will be also very interesting to study the mechanisms that are underlying this increased locomotor activity, which might be attributable to NF-κB’s role for the brain [39].

In summary, most of the reports show a stunning biological variability of the p65 knock-in phenotypes rather than a uniform modulation of NF-κB-mediated transcription. We anticipate that one DNA-binding subunit can be simultaneously modified at several positions, but the typical modification patterns must be revealed in future studies. The analysis of further p65 variants that are simultaneously mutated at several sites will probably reveal a more complete picture of the regulatory potential of the auxiliary NF-κB regulating pathways.

## 5. The Multiple Roles of NF-κB in Cancer

The central position of NF-κB as a signaling hub in human physiology also involves this network in several human ailments, including cancer. A Pubmed search with the keywords “NF-κB” and “cancer” revealed >17,200 Pubmed entries for this topic. The complex role of NF-κB in cancer can be attributed to changes in the canonical and noncanonical pathways, and to indirect and direct mechanisms.

The direct contribution of the NF-κB system to cancer was revealed by a number of genetic changes of NF-κB regulators detected in cancer cells [40]. These changes include mutation, amplification, and fusion of NF-κB regulators. Constitutive or chronic NF-κB activation can be achieved by gain-of-function (GOF) mutations that lead to continuous signaling to the IKK complex. Alternatively, the same effect can be achieved by loss-of-function (LOF) mutations disrupting negative regulators, such as CYLD or A20, which can lead to the inappropriate termination of the NF-κB response. The indirect contribution of NF-κB is attributable to the fact that this transcription factor contributes to the regulation of most (but not all) classical hallmarks of cancer, as defined by the Hanahan and Weinberg review: sustained proliferative signaling, resistance to cell death, replicative immortality, induction of angiogenesis, activation of invasion and metastasis, and reprogramming of energy metabolism [41]. But, also enabling characteristics, such as the acquisition of genome instability and tumor-promoting inflammation, are processes in which NF-κB-is involved.

### 5.1. The Indirect Roles of NF-κB in Cancer

**Figure 3:**
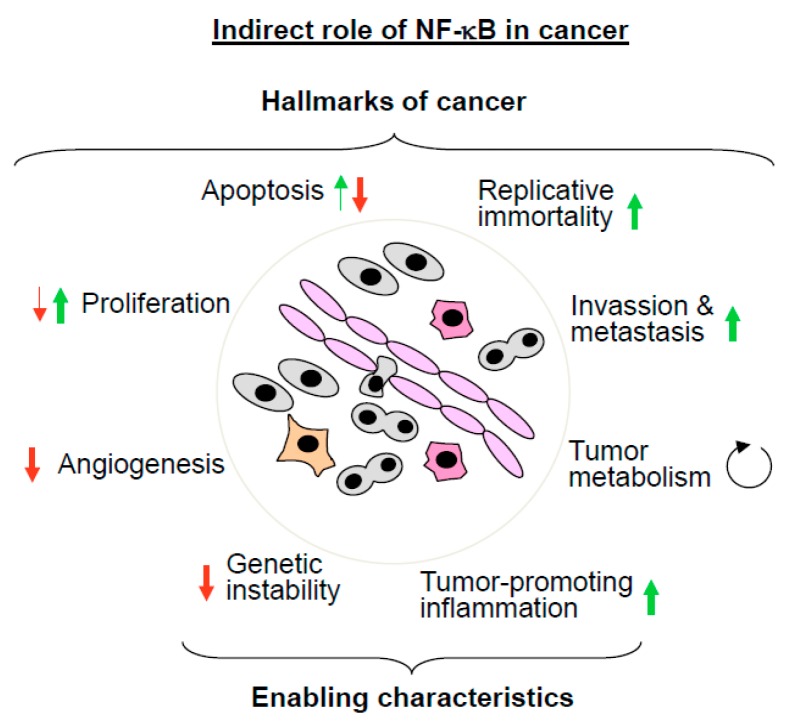
Schematic summary of the indirect roles of NF-κB for the hallmarks and enabling characteristics of cancer. The relative thickness of the arrows indicates the estimated relative contribution of NF-κB for the support (arrow up) or antagonism (arrow down) for the indicated processes.

In this section we will discuss the indirect roles of NF-κB in cancer by describing its contribution to the classical hallmarks and enabling characteristics, as schematically depicted in Figure 3. Many apoptotic stimuli, including the cytokine TNF, ionizing radiation, and chemotherapeutic agents, such as daunorubicine, trigger the anti-apoptotic functions of NF-κB [42,43,44]. This mechanism is clinically important as many chemotherapeutical agents induce NF-κB, which consequently protects cancer cells from cell death. This phenomenon has been described in numerous studies, for example in cisplatin and camptothecin-treated lung cancer [45] and anthracycline- and taxane-treated breast cancer [46]. These results led to the suggestion that targeting the anti-apoptotic role of NF-κB helps to overcome therapy resistance [47]. Proof-of-concept studies showed that selective NF-κB inhibition enhances the response to chemotherapy in gastric cancer [48] and IKK inhibition sensitizes melanoma cells to doxorubicin-induced cell death [49]. Of note, in some settings, NF-κB can also trigger cell death, for example in response to oxidative stress [50] or in the execution of TRAIL- and CD95-mediated apoptosis [51].

The role of NF-κB for replicative immortality is not well established, but an extensive crosstalk between the telomerase catalytic subunit (TERT) and NF-κB has been described. TERT activity ensures telomere elongation and thus helps to evade tumors from the fate of replicative senescence. NF-κB p65 was found to modulate TNF-induced nuclear translocation as well as telomerase activity of TERT in multiple myeloma cells [52]. The expression of the *Tert* gene is also directly upregulated by NF-κB [53]. But, this regulation is mutual, as vice versa TERT directly regulates NF-κB-dependent gene expression by binding to DNA-bound p65, potentially leading to hyperexpression of an extremely tumor promoting set of genes [54].

Also, the activation of invasion and metastasis involves NF-κB-dependent processes. NF-κB plays a significant role in the regulation of epithelial-mesenchymal transition (EMT), which is an early event in metastasis [55]. Inhibition of NF-κB signaling by expression of a dominant negative IκBα prevents EMT in Ras-transformed epithelial cells. This study also showed that NF-κB activation increases the transition to a mesenchymal phenotype, while its inhibition in mesenchymal cells causes even a reversal of EMT [56]. Collectively, these data suggest that NF-κB contributes both to the induction and also the maintenance of EMT. TNF-triggered EMT depends on NF-κB-mediated upregulation of Twist1, which is one of the key transcription factors modulating EMT [57]. In breast cancer cells, NF-κB activation contributes not only to expression of Twist1, but also to further EMT-regulating transcription factors, such as SLUG and SIP1 (Smad interacting protein 1) [58]. In addition, many cell adhesion molecules, such as integrins, selectins, ICAM-1 (intercellular adhesion molecule 1), E-selectin, and VCAM-1 (vascular cell adhesion molecule 1) are directly regulated by NF-κB [59]. These molecules contribute to cancer cell extravasation, but NF-κB activity is also important in the non-tumorigenic cells at the remote sites, which are colonized by tumor cells. This was shown in a mouse model of metastasis where the injection of lung carcinoma cells results in a reduced formation of metastatic foci in the livers of animals with a liver-specific deletion of IKKβ [60].

More recent evidence showed a contribution of NF-κB for the remodeling of tumor metabolism. Tumor cells often (but not always) prefer to use glycolysis instead of oxidative phosphorylation, as initially reported in 1927 by Otto Warburg [61]. The preferential use of glycolysis leads to a reduced ATP production, but might allow tumor cells to survive under hypoxic conditions and also to deliver substrates and intermediates for various anabolic pathways [62]. The shift from oxidative phosphorylation to glycolysis is mainly mediated by p53 and hypoxia inducible factor (HIF)-1, but also NF-κB participates in this regulation. Knockdown of NF-κB p65 in mouse embryonic fibroblasts causes cellular reprogramming to aerobic glycolysis, thus recapitulating the Warburg effect [63]. After glucose starvation, NF-κB p65 triggers p53 expression, which in turn leads to the mitochondrial upregulation of cytochrome c oxidase 2 [63], which is a crucial component of the electron transport chain. Another report showed that loss of p53 leads to p65-triggered expression of the glucose transporter GLUT3 (glucose transporter 3) in tumor cells, resulting in increased glucose consumption and anaerobic glycolysis [64]. The p65 protein can also be transported into the mitochondria in the absence of p53. Mitochondrial p65 can associate with the mitochondrial genome to repress mitochondrial gene expression and oxidative phosphorylation [65], and it will be interesting to study the molecular mechanisms allowing p65 entry into the mitochondria.

NF-κB also sustains proliferative signaling, but this is seen only in specific cell types, such as lymphocytes. An example for a direct and cell-specific effect of NF-κB is provided by mice that are lacking the DNA-binding subunit c-Rel, which display defects in B-cell proliferation [66,67]. Similarly, also RelB-deficient mice show defective proliferative responses in B cells [68]. In addition, *Rel^-/-^* mice show reduced keratinocyte proliferation and epidermal thickness [69]. Growth inhibitory roles of NF-κB have also been reported in mice expressing a dominant-negative IκBα protein. These mice show hyperplasia of the epidermal epithelium, suggesting that NF-κB restricts the proliferation of this cell type in vivo [70]. In addition to its importance for proliferation of specific cell types, NF-κB might also be relevant for proliferation of cancer cells, although this aspect is not fully explored. For example, RelB is required for proliferation of endometrioid adenocarcinoma [71] and overexpression of dominant-negative IκBα interferes with the proliferation of cervix carcinoma cells [72].

Many solid tumors induce angiogenesis in order to ensure the supply of nutrients and oxygen as well as to evacuate carbon dioxide and metabolic wastes [41]. This process is antagonized by NF-κB, as revealed in a study using mice expressing a dominant-negative IκBα protein exclusively in endothelial cells, resulting in the selective repression of NF-κB in endothelial cells. Inoculated tumors grow faster and more aggressive in these transgenic mice, concomitant with a striking increase in tumor vascularization [73]. Therefore, angiogenesis repression by NF-κB challenges the paradigm that systemic NF-κB inhibition can serve as a universal anti-cancer strategy.

But, NF-κB also regulates two cancer enabling characteristics of cancer cells. The contribution of NF-κB for tumor-promoting inflammation is very well documented and also covered by a number of excellent reviews [74,75]. In order avoid repetition and redundancy, we refer to these articles, which convincingly summarize how cells of the immune system create a tumor-promoting microenvironment by the NF-κB-dependent production of growth and survival factors.

The enabling characteristics of cancer include the acquisition of genetic instability, which confers selective advantage on cell subclones to enable their outgrowth and succession of clonal expansions [41]. NF-κB is activated by DNA damage, for example, by an ATM-mediated pathway in response to DNA double strand breaks induced by oxidative stress. In addition, NF-κB also contributes to the repair of double-strand breaks by homologous recombination. DNA damage triggers the association of p65 with the CtIP-BRCA1 complex and thus stimulates DNA repair [76]. A role of NF-κB for genome stability was revealed by the analysis of p65-deficient mouse and human cells, which show all of the signs of genomic instability, including high frequencies of gene deletions, DNA mutations, and chromosomal translocations [77]. The molecular mechanisms explaining the contribution of p65 for genomic stability are not clear and need to be elucidated in the future.

In summary, numerous studies have revealed an important contribution of NF-κB to many aspects of cancer biology. Although the majority of features are consistent with a tumor-promoting function of this transcription factor, a global inhibition of NF-κB is not possible, as it would foster angiogenesis and genetic instability.

### 5.2. The Direct Roles of NF-κB in Cancer

**Figure 4:**
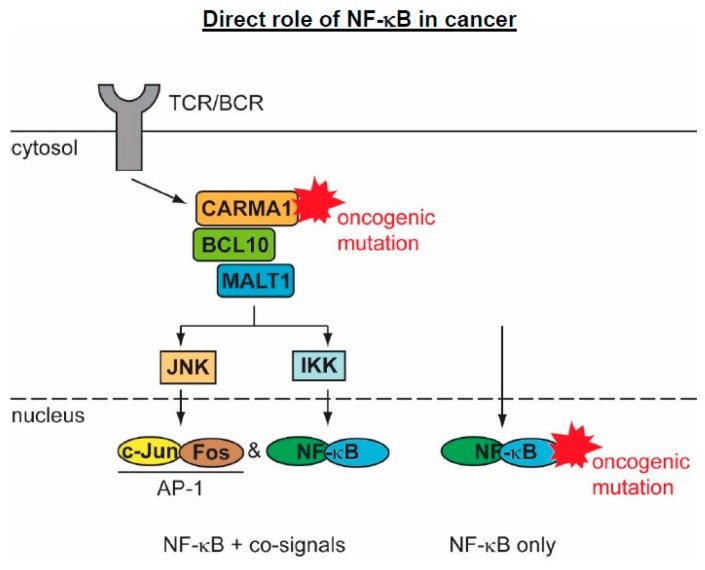
Schematic display of mutations occurring at upstream or downstream components of the NF-κB system. Mutations of upstream regulators are exemplified by the gain-of-function (GOF) mutations in the CBM complex that typically lead to activation of further tumor-promoting signaling pathways.

The direct contribution of the NF-κB system to cancer is seen by the direct mutation of NF-κB regulatory proteins [40]. Many of these mutations lead to lymphomas, which likely have its cause in the important role of the NF-κB pathway in normal lymphocyte development and activation. A review by Neil Perkins makes the important point that single mutations in NF-κB DNA-binding subunits are relatively rare, as they cannot adequately mimic the diverse nature and complexity of the NF-κB response [78]. Thus, the majority of NF-κB-driven cancers are induced by gain-of-function (GOF) mutations in the upstream activators of NF-κB. While GOF mutations of upstream activators lead to tumor formation that is dependent on the simultaneous induction of further signaling pathways, mutations of the DNA-binding subunits can trigger oncogenesis without the requirement for additional signals, as summarized in Figure 4.

The contribution of NF-κB for tumorigenesis by upstream activators is exemplified by mutations that are found in components of the so-called CBM complex, which is essential for NF-κB activation in lymphocytes by activation of the T-cell receptor (TCR) or B-cell receptor (BCR) [79]. The CBM complex is composed of the scaffold protein CARMA1/CARD11 (CARD-MAGUK1/caspase recruitment domain family member 11), the adaptor protein BCL10 (B-Cell CLL/lymphoma 10) and MALT1 (mucosa-associated lymphoid tissue lymphoma translocation protein 1), which has a dual role as a scaffold protein and a paracaspase [80,81].

Receptor activation leads to the recruitment of CARMA1 to lipid rafts, followed by its phosphorylation, which induces a conformational change, thus allowing for its oligomerization, the formation of BCL10 filaments and the attachment of MALT1 [82]. The BCL10/MALT1 filaments are further decorated by the ubiquitin E3 ligase TRAF6, which likely results in the all-or-none activation of the downstream pathways [82]. Active TRAF6 mediates the attachment of K63-branched polyubiquitin chains at the C-terminus of MALT1, which allows for further protein/protein interactions and the activation of NF-κB and JNK/AP1 (c-Jun N-terminal kinase/activator protein 1) signaling. The MALT1 protein has proteolytic activity and cleaves its substrate proteins after a positively charged arginine residue in the P1 position [80,81]. Mutations leading to the constitutive activation of the CBM complex are frequently found in lymphoid malignancies. GOF mutations of CARMA1 often occur in diffuse large B-cell lymphoma, B-cell lymphoproliferative disorders, and also in T-cell leukemia/lymphomas [83,84]. But, also the other members of the CBM complex are frequently found to be mutated, overexpressed, or affected by chromosomal translocations (MALT1-API2 or c-IAP2-MALT1). The GOF mutations of CBM members promote lymphomagenesis and MALT1 inhibitors have shown to allow for new treatment options for patients with refractory t(11;18)-positive MALT1 lymphoma [85,86,87]. The constitutively active CBM complex leads to constant activation of NF-κB and also to the permanent induction of the JNK/AP1 pathway and expression of RNA-binding proteins (Roquin-1 and -2, Regnase-1), which mediate the stabilization of several NF-κB-dependent transcripts. Of note, constitutive activation of NF-κB alone is not sufficient for lymphomagenesis and requires further JNK-derived signals. This was revealed in a mouse model where the transgenic expression of the constitutively active CARMA1 mutant CARMA1 (L225LI) is sufficient to trigger aggressive B-cell lymphoproliferation. Inhibition of either NF-κB or JNK blocks proliferation of the CARMA1 (L225LI)-expressing B-cells, showing that only cooperative NF-κB and JNK activation drives the malignant growth [88].

**Figure 5:**
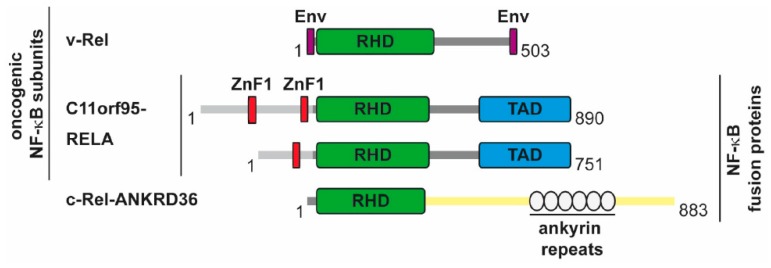
Schematic summary of oncogenic NF-κB DNA-binding subunits. The color code is similar to Figure 1, the v-Rel protein contains short sequence remnants from the REV env (envelope) gene at the N- and C-termini. Different parts of the C11ORF95 proteins, which contain zinc fingers (ZnF) are fused to the N-termini of p65.

While mutation of the upstream activators of NF-κB necessarily affects the co-regulatory pathways, there is also evidence for an oncogenic function of the DNA-binding subunits, as summarized in Figure 5. The DNA-binding subunits represent the endpoint of the NF-κB cascade and their ability to mediate oncogenic transformation shows the importance of NF-κB-dependent transcriptional programs. Soon after cloning of the avian retroviral protein v-Rel it became clear that retroviruses encoding v-Rel are highly oncogenic and transform chicken cells in vitro and in vivo [89,90]. Transgenic mice expressing v-Rel in T-cells develop immature, multicentric aggressive T-cell leukemia/lymphomas, showing that ectopic expression of v-Rel alone is sufficient for oncogenic transformation [91]. Constitutive p50/v-Rel DNA-binding is induced upon v-Rel epression, but the transforming activity also occurs in transgenic thymocytes lacking p50. The ability of v-Rel to trigger or repress gene expression depends on the cell type [92], but v-Rel-mediated induction of gene expression seems not to be as potent as gene induction by p65 [93]. Transformed chicken spleen cells show v-Rel localization in the nucleus and also in the cytoplasm. [94], but a threshold nuclear level of the v-Rel oncoprotein is required for the transformation of avian lymphocytes [95]. In the nucleus, v-Rel cooperates with many transcription factors, such as the AP1 subunits c-Fos and c-Jun, IRF4, and SP1 to mediate oncogenic transformation [96,97,98].

From all five different DNA-binding subunits, only the v-Rel homologue c-Rel has the ability to transform avian lymphoid cells dependent from the presence of both C-terminal TADs [99,100]. However, the removal of either of the 2 C-terminal TADs from c-Rel augments its transforming activity, suggesting that a chronic low-level of transcriptional activation is optimal for oncogenic transformation [101]. The c-Rel encoding *REL* locus is frequently altered (rearranged, amplified, mutated) in a variety of B- and T-cell malignancies. The *REL* locus at the chromosomal position 2p16.1-15 is amplified in Hodgkin’s lymphoma (~46%) and diffuses large B cell lymphoma (DLBCL) (~15%) [101]. Also, T-cell lymphomas (natural killer, peripheral T cells, anaplastic large cells) show the frequent amplification of the *REL* locus [102]. Increased c-Rel expression has been observed in Hodgkin lymphoma where the *REL* locus was found to be translocated to a position near the light chain enhancer [103], or alternatively by integration of an EBV genome near to the c-Rel encoding region [104]. Experimental proof for a causative role of c-Rel overexpression in the process of tumorigenesis has been obtained in a mouse model. Overexpression of c-Rel under the control of a hormone-responsive mouse mammary tumour virus promoter led to the induction of mammary tumors, but these develop with a long latency, suggesting the requirement of secondary events [105]. Also, nuclear localisation of c-REL is frequently observed in cancer cells. 

Furthermore, oncogenic fusion proteins have been described for c-Rel and p65. A chimeric c-REL-ANKRD36 protein occurs in the RC-K8 DLBCL cell line due to a deletion on chromosome 2 [106]. Interestingly, the fusion partner ANKRD36 has several ankyrin repeats, which are also found in p100, p105 and the IκB proteins. However, it is unclear whether this fusion protein is responsible for the proliferation of the RC-K8 cells. Another oncogenic fusion protein, C11orf95-RELA, is expressed in more than two-thirds of supratentorial ependymomas, a rare brain tumor, where C11orf95-RELA accumulates in the nuclei [107]. A causative role of C11orf95-RELA for tumorigenesis was shown in experiments where the expression of the fusion protein in mouse forebrain neural stem cells converted them to brain tumor cells, probably by driving an aberrant transcription program [107]. The various C11orf95-RELA fusion proteins contain one or two zinc finger domains, raising the possibility that this fusion can alter the DNA-binding specificity of NF-κB. It will thus be interesting to characterize the genomic binding sites of C11orf95-RELA and to compare them to the binding sites of the wildtype p65 protein in healthy tissue. The finding that C11orf95-RELA expression generates only one highly specific cancer type also illustrates the high plasticity and diversity of the NF-κB response.

## 6. Concluding Remarks

The NF-κB transcription factor system is of broad relevance for the formation and maintenance of many tumor entities. Its broad relevance for central physiological processes does not allow for its global inhibition and raises the need for the development of highly pathway-specific and cell-type specific components as exemplified by MALT1 inhibitors, which are currently tested in clinical trials for the treatment of lymphomas. Further work is needed in order to identify and characterize the auxiliary components that help to specify and regulate the transcriptional output programs of NF-κB, as these proteins could be additional druggable targets.
