# Correction: Riedlinger, T. et al. The Direct and Indirect Roles of NF-κB in Cancer: Lessons from Oncogenic Fusion Proteins and Knock-In Mice. *Biomedicines*, 2018, *6*, 36

**DOI:** 10.3390/biomedicines6020057

**Published:** 2018-05-16

**Authors:** Tabea Riedlinger, Jana Haas, Julia Busch, Bart van de Sluis, Michael Kracht, M. Lienhard Schmitz

**Affiliations:** 1 Institute of Biochemistry, Justus-Liebig-University, D-35392 Giessen, Germany; Tabea.Riedlinger@biochemie.med.uni-giessen.de (T.R.); Jana.Haas@biochemie.med.uni-giessen.de (J.H.); Julia.Busch@biochemie.med.uni-giessen.de (J.B.); 2 Department of Pediatrics, Molecular Genetics Section, University of Groningen, University Medical Center Groningen, Antonius Deusinglaan 1, 9713 AV, Groningen, The Netherlands; a.j.a.van.de.sluis@umcg.nl; 3 Rudolf-Buchheim-Institute of Pharmacology, Justus-Liebig-University, D-35392 Giessen, Germany; Michael.Kracht@pharma.med.uni-giessen.de

We would like to report an error in a previously published paper [1]. The details are as follows:

Please note that Figure 1 contains a mistake, as we erroneously indicate p105/p52 instead of p105/p50, and p100/p50 instead of p100/p52.

Please replace this figure: 
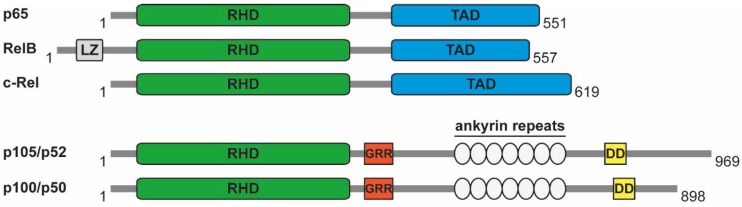
 with the following:

**Figure 1:**
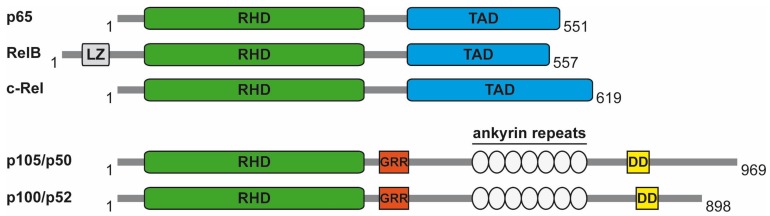
DNA-binding subunits of NF-κB. The functional domains of the five DNA-binding subunits, including the leucine zipper (LZ), the glycine-rich region (GRR), and the death domain (DD) are shown. The number of amino acids is provided for human proteins.

These changes have no material impact on the conclusions of our paper. The authors would like to apologize for any inconvenience caused to the readers by these changes.
